# WHO European Childhood Obesity Surveillance Initiative: School Nutrition Environment and Body Mass Index in Primary Schools

**DOI:** 10.3390/ijerph111111261

**Published:** 2014-10-30

**Authors:** Trudy M.A. Wijnhoven, Joop M.A. van Raaij, Agneta Sjöberg, Nazih Eldin, Agneta Yngve, Marie Kunešová, Gregor Starc, Ana I. Rito, Vesselka Duleva, Maria Hassapidou, Éva Martos, Iveta Pudule, Ausra Petrauskiene, Victoria Farrugia Sant’Angelo, Ragnhild Hovengen, João Breda

**Affiliations:** 1 Division of Noncommunicable Diseases and Life-Course, WHO Regional Office for Europe, UN City, Marmorvej 51, DK-2100 Copenhagen ø, Denmark; E-Mail: jbr@euro.who.int; 2 Centre for Nutrition, Prevention and Health Services, National Institute for Public Health and the Environment, P.O. Box 1, 3720 BA Bilthoven, The Netherlands; E-Mail: joop.van.raaij@rivm.nl; 3 Division of Human Nutrition, Wageningen University, P.O. Box 8129, 6700 EV Wageningen, The Netherlands; E-Mail: joop.vanraaij@wur.nl; 4 Department of Food and Nutrition and Sport Science, University of Gothenburg, P.O. Box 300, SE-405 30 Gothenburg, Sweden; E-Mail: agneta.sjoberg@gu.se; 5 Health Promotion Department, Health Service Executive, Railway Street, Navan, County Meath, Ireland; E-Mail: nazih.eldin@hse.ie; 6 National Nutrition Surveillance Centre, School of Public Health, Physiotherapy & Population Science, University College Dublin, Belfield, Dublin 4, Ireland; 7 School of Hospitality, Culinary Arts and Meal Science, Örebro University, Campus Grythyttan, P.O. Box 1, SE-712 60 Grythyttan, Sweden; E-Mail: agneta.yngve@oru.se; 8 Obesity Management Centre, Institute of Endocrinology, Narodni 8, 11694 Prague 1, Czech Republic; E-Mail: mkunesova@endo.cz; 9 Faculty of Sport, University of Ljubljana, Gortanova 22, 1000 Ljubljana, Slovenia; E-Mail: gregor.starc@guest.arnes.si; 10 National Health Institute Doutor Ricardo Jorge, Av. Padre Cruz, 1649-016 Lisbon, Portugal; E-Mail: ana.i.rito@gmail.com; 11 Department of Food and Nutrition, National Centre of Public Health and Analyses, 15 Akad. Ivan Evstatiev Geshov Blvd., 1431 Sofia, Bulgaria; E-Mail: v.duleva@ncpha.government.bg; 12 Department of Nutrition and Dietetics, Alexander Technological Educational Institute of Thessaloniki, P.O. Box 14561, 54101 Thessaloniki, Greece; E-Mail: mnhas@nutr.teithe.gr; 13 National Institute for Food and Nutrition Science, Albert Florian Út 3/a, 1097 Budapest, Hungary; E-Mail: martos.eva@oeti.antsz.hu; 14 Centre for Disease Prevention and Control, 22 Duntes Street, LV-1005 Riga, Latvia; E-Mail: iveta.pudule@spkc.gov.lv; 15 Department of Preventive Medicine, Lithuanian University of Health Sciences, Eiveniu Str. 4, 50009 Kaunas, Lithuania; E-Mail: ausrapet@vector.kmu.lt; 16 Primary Health Care Department, 7 Harper Lane, Floriana FLR 1940, Malta; E-Mail: victoria.farrugia-santangelo@gov.mt; 17 Department of Health Statistics, Norwegian Institute of Public Health, P.O. Box 4404, Nydalen, N-0403 Oslo, Norway; E-Mail: ragnhild.hovengen@fhi.no

**Keywords:** school policy, monitoring, healthy school environment, nutrition, physical activity, overweight, primary schools, Europe

## Abstract

*Background:* Schools are important settings for the promotion of a healthy diet and sufficient physical activity and thus overweight prevention. *Objective:* To assess differences in school nutrition environment and body mass index (BMI) in primary schools between and within 12 European countries. *Methods*: Data from the World Health Organization (WHO) European Childhood Obesity Surveillance Initiative (COSI) were used (1831 and 2045 schools in 2007/2008 and 2009/2010, respectively). School personnel provided information on 18 school environmental characteristics on nutrition and physical activity. A school nutrition environment score was calculated using five nutrition-related characteristics whereby higher scores correspond to higher support for a healthy school nutrition environment. Trained field workers measured children’s weight and height; BMI-for-age (BMI/A) Z-scores were computed using the 2007 WHO growth reference and, for each school, the mean of the children’s BMI/A Z-scores was calculated. *Results*: Large between-country differences were found in the availability of food items on the premises (e.g., fresh fruit could be obtained in 12%−95% of schools) and school nutrition environment scores (range: 0.30−0.93). Low-score countries (Bulgaria, Czech Republic, Greece, Hungary, Latvia and Lithuania) graded less than three characteristics as supportive. High-score (≥0.70) countries were Ireland, Malta, Norway, Portugal, Slovenia and Sweden. The combined absence of cold drinks containing sugar, sweet snacks and salted snacks were more observed in high-score countries than in low-score countries. Largest within-country school nutrition environment scores were found in Bulgaria, Czech Republic, Greece, Hungary, Latvia and Lithuania. All country-level BMI/A Z-scores were positive (range: 0.20−1.02), indicating higher BMI values than the 2007 WHO growth reference. With the exception of Norway and Sweden, a country-specific association between the school nutrition environment score and the school BMI/A Z-score was not observed. *Conclusions*: Some European countries have implemented more school policies that are supportive to a healthy nutrition environment than others. However, most countries with low school nutrition environment scores also host schools with supportive school environment policies, suggesting that a uniform school policy to tackle the “unhealthy” school nutrition environment has not been implemented at the same level throughout a country and may underline the need for harmonized school policies.

## 1. Introduction

Overweight and obesity among children and adolescents remain as public health problems in the European Region of the World Health Organization (WHO). Excess body weight in childhood and adolescence is associated with a higher risk of premature death and disability in adulthood, but overweight children and adolescents are also more likely to develop noncommunicable diseases such as diabetes at a younger age [1,2]. These problems were recognized by and discussed between European Member States for the first time at the WHO European Ministerial Conference on Counteracting Obesity in 2006 [3] and were given follow-up at the WHO European Ministerial Conference on Nutrition and Noncommunicable Diseases in the context of Health 2020 in 2013 [4]. The European Union (EU) Action Plan on Childhood Obesity, which was launched during the Greek Presidency of the EU in February 2014, identifies children as the priority targets for action [5].

The establishment of the WHO European Childhood Obesity Surveillance Initiative (COSI) as a response to the 2006 Ministerial Conference has been the start of population-based monitoring at regular intervals of overweight and obesity among primary-school children in the Region. COSI aims to measure in a standardized way children’s weight and height in order to monitor the progress in reducing overweight and to allow intercountry comparisons within this population group [6]. With the development of the COSI protocol, it was stressed that it could also be merged with other protocols to evaluate the impact of preventive interventions in school settings [7,8]. Thirteen Member States participated in the first round of measurements in school year 2007/2008 and, since then, more countries have joined this initiative.

The imbalance between energy intake and energy expenditure (including for physical activity) explains to a large extent the current overweight epidemic [2]. Individual energy intake and expenditure are affected by a wide range of environmental influences, including the obesogenic school environment [2,9,10,11,12]. School settings can be important settings to promote healthy lifestyles, in conjunction with a whole-of-society approach involving the local community and addressing health inequity. For example, schools may influence children’s diets by providing school meals, participating in school fruit schemes such as those of the EU [13], controlling the availability of foods and non-alcoholic beverages and including nutrition education in the curriculum. In addition, schools can also be important settings for the promotion of physical activity, for example, through the inclusion of physical education lessons in the curriculum, better equipped play grounds, promotion of unorganized activity during breaks and the use of outdoor environments in teaching different subjects [14,15,16,17,18,19,20].

It would be useful if schools could be characterized in terms of their contribution to the “obesogenic” environment so that policy-makers and schools can take this into account when they plan to implement new or strengthen existing school policies for a healthier school environment. An assessment would be key not only to improve school-based interventions but also to monitor national policy implementation on childhood obesity. COSI also includes a school form that involves the collection of information on some school environmental characteristics related to nutrition and physical activity. It is envisaged that the information gathered by the school form on the nutrition and physical activity environment in primary schools along with the results of the mandatory height and weight measurements [6], may assist schools in developing a prevention strategy or intervention programme based on a supportive environment, with the aim of promoting healthy choices on their premises.

The inter-country analyses on body mass index (BMI) and the prevalence of overweight in both rounds suggested the presence of a north-south gradient with the highest level of overweight found in southern European countries [21,22]. In the present study, we aimed to: (1) assess regional differences within Europe and variability within a country in primary schools with respect to the availability of foods and beverages on their premises; (2) apply proxy indicators to distinguish schools in terms of their nutrition environment and in terms of BMI, by using the COSI data of two rounds (2007/2008 and 2009/2010).

## 2. Methods

### 2.1. COSI Project

The first COSI data collection round took place from September 2007 to December 2008, with 13 countries participating: Belgium (Flemish region only), Bulgaria, Cyprus, Czech Republic, Ireland, Italy, Latvia, Lithuania, Malta, Norway, Portugal, Slovenia and Sweden. The second round was conducted from September 2009 to December 2010, with four new countries participating—Greece, Hungary, Spain and the former Yugoslav Republic of Macedonia—and two initial countries dropping out (Bulgaria and Sweden). Countries decided on the actual measurement period within the data collection rounds. Data collection, however, was avoided during the first two weeks of a school term or immediately after a major holiday [21,22].

The COSI protocol [7,8] is in accordance with the international ethical guidelines for biomedical research involving human subjects [23]. Depending on local circumstances, the procedures were approved by local ethical committees as well. Parents were fully informed about all study procedures, and informed consent was obtained. Children's consent was always obtained prior to the anthropometric measurements, and confidentiality of all collected and archived data was ensured [21,22]. A more detailed description of the implementation characteristics of both COSI rounds can be found elsewhere [6].

### 2.2. Sampling of Schools and Children

Nationally representative samples of children from all countries were included whereby the calculated sample size of ~2800 children per age group was based on an 80% power to detect a minimum difference of 0.10 Z-score in mean BMI per year at a two-sided 5% significance level and applying a design effect of 1.2. According to the first consultation in 2007 with the participating countries, an average of ~25 pupils per class was assumed. Taking into account a consent rate of 80% and 90%, respectively, ~124/140 classes would be required to achieve the final recommended sample size of ~2800 pupils per targeted age group. Extra classes were required if there were fewer than 25 pupils or when there were lower attendance rates than expected [21,22].

The sample of children from Malta included all second grade classes in all 95 primary schools. The other countries applied cluster sampling using the primary school as Primary Sampling Unit (PSU) (except the Czech Republic where the PSU was composed of paediatric clinics because COSI was attached to the mandatory health checks performed by paediatricians). Primary schools were selected randomly with probability proportional to size from the list of all primary schools, which was centrally available in each country through the Ministry of Education or at the national school registry (or, as in the Czech Republic, the national list of primary care paediatricians). If all children of the specifically targeted age group were in the same grade, then one class per school was drawn within a grade level. If the specifically targeted age group was spread across grades, however, all grades where children from this age group were present could be sampled. Countries that participated in round 1 could decide for round 2 either to select a new nationally representative sample of schools or to use the same schools selected in round 1 and randomly select the classes from these sites, a “sentinel” approach. Four countries (Ireland, Lithuania, Norway and Portugal) used this sentinel site approach. Similar schools could have been selected by chance in round 2 by the countries that selected a new nationally representative sample of schools.

COSI targets 6-, 7-, 8- and 9-year-old children whereby countries could choose one or more of these four age groups. In round 1, all countries except Slovenia and Sweden targeted one COSI age group, whereas in round 2, all countries except Greece, Ireland, Lithuania and Slovenia targeted one age group. Detailed sampling characteristics have been described elsewhere [21,22]. In all countries except Hungary, all children registered in the sampled classes (regardless of whether they fell within the country’s targeted age range) who had informed parental consent and who were present on the survey day were approached to be measured. In Hungary only the children in the selected classes who fell within its targeted age group (7-year-olds) were measured.

Twelve out of the 17 COSI countries administered all mandatory school questions (see section 2.3) in round 1 or in round 2 and were included in the present analyses: Bulgaria, Czech Republic, Greece, Hungary, Ireland, Latvia, Lithuania, Malta, Norway, Portugal (data from four schools in Madeira, collected one year after the other Portuguese regions, were not taken into account in this paper), Slovenia and Sweden. Table S1 presents, for each country, the sampling characteristics of the schools and the median number of children measured per school. In total, 1831 schools in round 1 and 2045 schools in round 2 returned the school form. Schools that participated in the anthropometric measurements but did not return the school form were excluded from the analyses (n = 28). Table S2 gives, for each country, the data collection period of the anthropometric measurements and the period when the school form was completed.

### 2.3. School Nutrition Environment Score (Proxy Indicator)

The COSI school form included questions on the frequency of physical education lessons (including e.g., dancing) provided to the sampled class (minutes per week), the availability of school playgrounds (yes/no), the possibility to obtain food items and beverages on the school premises (15 items were listed; yes/no), and the organisation of school initiatives to promote a healthy lifestyle (e.g., to promote physical activity and/or healthy eating) by the sampled class (yes/no). These 18 characteristics were mandatory in the COSI school form and were considered as possible modifiable opportunities in school settings by national/local governments or by schools in favour of children’s healthy eating and physical activity patterns [7,8]. Moreover, they have been included in documents on the WHO framework for health promoting schools [19,20]. The school data were generally provided by the school principal, deputy headmaster or by the teachers involved with the sampled classes, except in the Czech Republic where the school form was filled in by the paediatrician or by a member of the study team together with the responsible person in the school.

Five out of the 18 characteristics under study were selected to calculate a school nutrition environment score because these items form an essential part of current policies at the European level or the national level. Two are included in current EU policies (school fruit scheme [13] and school milk programme [24]) to promote healthy eating habits and are considered supportive to a healthy school environment. Three characteristics tend to have a high content of saturated fats, free sugars or salt, are nutrient-poor and are major categories of food being advertised by the food and drink sector. They are considered unsupportive to a healthy school environment and their availability on the school premises should be limited [25]. A score of 0 or 1 was given to each answer on these five characteristics that could be obtained on the school premises: “*fresh fruit*”, “*milk*”, “*cold drinks containing sugar*”, “*sweet snacks*” and “*salted snacks*”. This grading was done according to the potential influence a school characteristic might have (to our best knowledge from literature) on the risk of excess body weight in children or its support to a healthy school environment [26,27,28,29,30,31]. An answer was graded with 1 when it was considered supportive to a healthy school nutrition environment (presence of fresh fruit and milk; absence of cold drinks containing sugar, sweet snacks and salted snacks) and with 0 when it was considered unsupportive to a healthy school nutrition environment (absence of fresh fruit and milk; presence of cold drinks containing sugar, sweet snacks and salted snacks). A school nutrition environment score was calculated for each school that provided information on all of them by dividing the total attained supportive scores (range: 0−5) by 5. A school score of 0.0 means that all characteristics were graded as unsupportive, and a score of 1.0 means that all characteristics were graded as supportive. Three schools in round 1 and two schools in round 2 did not provide information on all five selected characteristics and were excluded from these analyses.

### 2.4. School BMI-for-Age Z-Score (Proxy Indicator)

Taking into account the local arrangements and available budget, countries chose the most appropriate professionals to collect the children’s data (e.g., physical education teachers, nationally or regionally based health professionals) and trained them. The children’s body weights and heights were measured according to WHO standardized techniques [32]. Children were asked to take off their shoes and socks as well as all heavy clothing (coats, sweaters, jackets, *etc.*) and remove items such as wallets, mobile phones or key chains. Body weight was measured to the nearest 0.1 kg with portable digital (mainly manufacturer-calibrated) scales, and body height was measured standing upright to the nearest 0.1 cm with portable stadiometers. The average weights of types of clothing were provided by each country and were used to adjust the measured body weight for the weight of the clothes worn. BMI was calculated using the formula: adjusted weight (kg) divided by height squared (m^2^). The 2007 WHO BMI-for-age (BMI/A) distributions for schoolchildren were used to compute BMI/A Z-scores [33]. The BMI/A Z-scores dataset included children for whom informed consent was given, from whom complete information on age, sex, weight and height was available and who had biologically plausible values (a BMI/A Z-score between –5 and +5) [34]. Only 169 out of 83,678 children (0.20%) fell outside the plausibility range.

A school BMI/A Z-score was calculated as the mean of the children’s BMI/A Z-scores and was only calculated for those schools that had at least 15 children with complete information. The Czech Republic was excluded from this analysis because only 3% of their schools had measured more than 15 children. Three (Ireland, Lithuania and Slovenia) of the seven countries that participated in both rounds targeted an additional age group in round 2 (9-year-olds); the data on the 9-year-olds were not used in this analysis because there were no corresponding round 1 data to which the BMI/A Z scores could be compared. For the comparison of the school BMI/A Z-score between the rounds, the classes that were sampled to enrol these 9-year-olds in round 2 were deleted for this purpose.

### 2.5. Statistical Analyses

Descriptive analyses included the calculation of percentages for the 18 school characteristics and the examination of their differences across the countries by round using chi-squared tests. If the chi-squared tests were found significant, the Marascuilo procedure [35] was used for the multi-group comparisons of proportions between countries. Schools were categorized by the number of selected nutrition-related characteristics graded as supportive (range 0–5).

For each country-specific dataset, the Shapiro-Wilk test was used to assess whether the calculated school nutrition environment scores and the school BMI/A Z-scores were normally distributed. The results of this test as well as the results of separate tests on skewness and kurtosis revealed that parametric tests could be performed. In addition, mean, standard deviation (SD) and coefficient of variation (CV) of the school scores were calculated. The one-way analysis of variance (ANOVA) test was used to assess the significant difference of mean scores across the countries in each round, followed by the Games-Howell *post hoc* test for the multiple comparisons between countries [36]. A two-way ANOVA test was applied to assess the interaction effect of country and round on the mean scores in the seven countries that participated in both rounds. In the case of significant interaction effect, the unpaired t-test was performed to assess whether the difference in mean values between the two rounds in each of the seven countries was statistically significant. Univariate linear regression analyses were performed by country to test the association of the school BMI/A Z-score with school category as well as multiple linear regression analyses by round with country as covariate.

A *p* value of <0.05 was used to define statistical significance. All statistical analyses except the Games-Howell *post hoc* test were performed in Stata version 10.1 (StataCorp LP, College Station, TX, USA). The latter was performed in SPSS version 20.0 (IBM, Armonk, New York, NY, USA).

## 3. Results and Discussion

### 3.1. Results 

#### 3.1.1. School Environment Characteristics on Nutrition and Physical Activity

Eighty-six per cent of the 1831 eligible schools in round 1 and 74% of the 2045 eligible schools in round 2 provided information on all 18 school environment characteristics under study, whereby all Irish, Latvian and Maltese schools in round 1 and all Slovenian schools in round 2 did so. The two physical activity-related characteristics and the general characteristic on school projects to promote a healthy life style had the highest number of missing values (Table S3).

The proportion of schools that provided the possibility to obtain food items and beverages on their premises, outside playgrounds or inside play areas, physical education lessons of ≥60 min per week or organized school initiatives to promote a healthy lifestyle in round 1 is presented in Table 1, whereas Table 2 displays similar information for round 2. There was large variation between the countries in the availability of food items or beverages. For instance fresh fruit could be obtained in 22%–75% of schools in round 1 (Table 1) and in 12%–95% of schools in round 2 (Table 2). Cold drinks containing sugar could be obtained on the premises of 40% or more of schools in five countries in round 1 and in three countries in round 2. Milk could be obtained in 33%–95% of schools in round 1 (Table 1) and in 18%–96% of schools in round 2 (Table 2). The highest proportion of schools that provided sweet snacks on their premises was found in Bulgaria (77%), Lithuania (69%, round 1; 60% round 2), Hungary (51%), Latvia (51%, round 1; 49% round 2) and the Czech Republic (46%, round 1; 27% round 2). Fewer schools made salted snacks available on their premises than sweet snacks, but 37% of Hungarian schools (round 2) and 74% of Bulgarian schools (round 1) still provided them. Norway was the only country in both rounds that did not make cold drinks containing sugar, sweet snacks and salted snacks available to pupils on their school premises. The two physical activity-related characteristics were more uniform across the countries (ranging from 76% to 100%), while reported initiatives to promote a healthy lifestyle (with a focus on physical activity promotion and/or healthy eating) varied from 42% of schools in Bulgaria and Greece to 97% of schools in Latvia. The results of the multicountry comparisons of round 1 and round 2 are also presented in Table 1 and Table 2, respectively.

**Table 1:** Frequency of 18 school nutrition and physical activity environment characteristics included in the mandatory COSI school form in round 1 (2007/2008), by country *.

Characteristics	BGR	CZE	IRL	LVA	LTU	MLT	NOR	PRT	SVN	SWE
Number of schools (*n*)	179	548	154	190	155	95	127	176	118	89
Availability at school (%)										
*Food Items that Can be Obtained on the School Premises*										
1 Fresh fruit **^†^**	36.9 **^a,b^**	55.5 **^c^**	22.7 **^a^**	52.6 **^b,c^**	72.9 **^d,e^**	22.1 **^a^**	70.1 **^c,d,e^**	35.2 **^a,b^**	75.0 **^§,e^**	65.2 **^c,d,e^**
2 Vegetables **^†^**	17.9 **^a,b^**	48.2 **^c^**	3.9 **^d^**	40.5 **^c,e^**	80.7 **^f^**	11.6 **^a,d^**	48.0 **^c,e^**	25.6 **^a,b,e^**	36.4 **^b,c,e^**	88.8 **^f^**
3 100% fruit juice without sugar **^†^**	19.0 **^a,b^**	25.7 **^a,b^**	4.6 **^c^**	21.6 **^a,b^**	54.2 **^d^**	11.6 **^a,c^**	15.0 **^a,c^**	4.0 **^c^**	39.0 **^b,d^**	11.2 **^a,c^**
4 Fruit juice containing sugar **^†^**	69.3 **^a^**	51.6 **^b^**	4.6 **^c,d^**	48.4 **^b^**	63.2 **^a,b^**	7.4 **^c,d^**	0.0 **^c^**	5.7 **^c,d^**	19.5 **^d^**	1.1 **^c^**
5 Cold drinks without sugar **^†^**	33.0 **^a,b^**	43.3 **^a^**	2.6 **^c,d^**	36.3 **^a,b^**	49.0 **^a^**	3.2 **^c,d^**	0.0 **^§,c^**	1.1 **^c^**	42.4 **^a,b^**	20.2 **^b,d^**
6 Cold drinks containing sugar **^†^**	68.2 **^a^**	56.6 **^a,b^**	0.7 **^c^**	40.5 **^b^**	70.3 **^a^**	2.1 **^c^**	0.0 **^§,c^**	2.8 **^c^**	40.7 **^b^**	6.7 **^c^**
7 Hot drinks without sugar **^†^**	43.6 **^a^**	30.8 **^a^**	0.0 **^b^**	36.8 **^a^**	46.5 **^a^**	0.0 **^b^**	0.0 **^§,b^**	5.1 **^b^**	40.7 **^a^**	3.4 **^b^**
8 Hot drinks containing sugar **^†^**	57.5 **^a^**	55.7 **^a^**	0.0 **^b^**	42.6 **^a,c^**	87.1 **^d^**	1.1 **^b^**	0.0 **^§,b^**	3.4 **^b^**	28.0 **^c^**	5.6 **^b^**
9 Diet or “light” soft drinks **^†^**	27.4 **^a,b^**	18.6 **^a^**	0.0 **^c^**	37.9 **^b^**	14.2 **^a,d^**	3.2 **^c,d^**	0.0 **^§,c^**	0.6 **^c^**	26.5 **^§,a,b^**	3.4 **^c,d^**
10 Milk **^†^**	33.5 **^a^**	57.7 **^b^**	33.1 **^a^**	42.1 **^a,b^**	42.6 **^a,b^**	59.0 **^b,c^**	95.3 **^d^**	77.3 **^c,e^**	94.9 **^d^**	86.5 **^d,e^**
11 Flavoured milk **^†^**	21.8 **^a,b^**	54.0 **^c^**	1.3 **^d^**	15.8 **^a^**	18.1 **^a,b^**	2.1 **^d^**	38.6 **^b,c^**	35.8 **^b^**	23.7 **^a,b^**	12.5 **^§,a,d^**
12 Water **^†^**	67.6 **^a,b^**	54.2 **^a^**	46.1 **^a^**	57.4 **^a,b^**	76.8 **^b^**	47.4 **^a^**	99.2 **^c^**	55.1 **^a^**	44.1 **^a^**	95.5 **^c^**
13 Yoghurt **^†^**	37.4 **^a^**	54.7 **^b,c^**	13.0 **^d^**	37.4 **^a^**	70.3 **^b^**	11.6 **^d^**	15.8 **^d,e^**	21.0 **^a,d,e^**	34.8 **^a,c,e^**	25.8 **^a,d,e^**
14 Sweet snacks **^†^**	76.5 **^a^**	45.6 **^b^**	0.7 **^c^**	51.1 **^b,d^**	69.0 **^a,d^**	8.4 **^c^**	0.0 **^c^**	9.1 **^c^**	3.4 **^c^**	3.4 **^c^**
15 Salted snacks **^†^**	73.7 **^a^**	18.6 **^b^**	0.0 **^c^**	14.7 **^b,d^**	22.6 **^b^**	1.1 **^c^**	0.0 **^c^**	3.4 **^c,d^**	9.3 **^b,c,d^**	2.3 **^c^**
*Physical Activity-Related Characteristics*										
16 Availability of outside playgrounds or inside play areas where children can play during school breaks **^†^**	99.4 **^§,a^**	78.4 **^§,b^**	98.7 **^a,c^**	92.6 **^a,c,d^**	88.2 **^§,b,c^**	100.0 **^a^**	100.0 **^a^**	98.3 **^§,a,c^**	82.1 **^§,b,d^**	100.0 **^§,a^**
17 Provision of ≥60 min per week of physical education to pupils from participating classes **^†^**	99.4 **^§,a^**	97.9 **^§,a,b^**	92.9 **^a,b^**	99.5 **^a^**	99.3 **^§,a^**	83.2 **^b^**	92.9 **^§,a,b^**	90.7 **^§,a,b^**	100.0 **^a^**	76.2 **^§,b^**
*General Characteristic*										
18 Any initiatives/projects organized to promote a healthy lifestyle among pupils from participating classes **^†^**	42.4 **^§,a^**	87.7 **^§,b^**	88.3 **^b,c^**	96.8 **^c^**	54.2 **^§,a^**	89.5 **^b,c^**	62.0 **^§,a^**	64.3 **^§,a^**	61.5 **^§,a^**	89.7 **^§,b,c^**

Notes: * The country codes refer to the International Organization for Standardization (ISO) 3166-1 Alpha-3 country codes; **^†^** Statistically significant difference of proportions across the ten countries in COSI round 1 (2007/2008) (chi-squared test; *p* < 0.001); **^§^** Not all schools that returned a school form provided information for this characteristic (Table S3); **^a,b,c,d,e,f^** Within each characteristic (*i.e.*, by row), proportions that share the same letter superscript do not statistically significantly differ from each other (Marascuilo procedure); BGR, Bulgaria; COSI, Childhood Obesity Surveillance Initiative; CZE, Czech Republic; IRL, Ireland; LTU, Lithuania; LVA, Latvia; MLT, Malta; NOR, Norway; PRT, Portugal; SVN, Slovenia; SWE, Sweden.

**Table 2:** Frequency of 18 school nutrition and physical activity environment characteristics included in the mandatory COSI school form in round 2 (2009/2010), by country *.

Characteristics	CZE	GRC	HUN	IRL	LVA	LTU	NOR	PRT	SVN
Number of schools (*n*)	882	123	98	154	169	160	125	167	167
Availability at school (%)									
*Food Items that Can be Obtained on the School Premises*									
1 Fresh fruit **^†^**	47.1 **^a^**	12.2 **^b^**	82.7 **^c,d^**	24.0 **^b^**	53.9 **^a,e^**	67.5 **^c,e^**	67.2 **^c,e^**	64.1 **^c,e^**	95.2 **^d^**
2 Vegetables **^†^**	39.0 **^a^**	4.9 **^b^**	23.5 **^a^**	4.6 **^b^**	44.4 **^a,c^**	72.5 **^d^**	37.6 **^a,c^**	27.0 **^a^**	56.9 **^c,d^**
3 100% fruit juice without sugar **^†^**	18.3 **^a^**	46.3 **^b,c^**	29.6 **^a,b^**	7.1 **^d,e^**	20.1 **^a,d^**	63.1 **^c^**	17.6 **^a,d^**	2.4 **^e^**	22.2 **^a,d^**
4 Fruit juice containing sugar **^†^**	30.7 **^a^**	32.5 **^a,b^**	52.0 **^b,c^**	4.6 **^d^**	47.3 **^b,c^**	59.4 **^c^**	0.0 **^d^**	3.6 **^d^**	35.9 **^a,b^**
5 Cold drinks without sugar **^†^**	27.7 **^a^**	12.2 **^b,c^**	28.6 **^a,b^**	1.3 **^c,d^**	34.3 **^a^**	42.5 **^a^**	0.0 **^§,d^**	1.8 **^c,d^**	28.1 **^a,b^**
6 Cold drinks containing sugar **^†^**	35.8 **^a^**	11.4 **^b,c^**	49.0 **^a,d^**	0.0 **^e^**	42.0 **^a^**	68.1 **^d^**	0.0 **^§,e^**	1.8 **^b,e^**	28.7 **^a,c^**
7 Hot drinks without sugar **^†^**	19.7 **^a^**	6.5 **^b,c^**	17.4 **^a,b^**	0.7 **^c^**	37.9 **^d^**	50.0 **^d^**	0.0 **^§,c^**	3.6 **^b,c^**	31.7 **^a,d^**
8 Hot drinks containing sugar **^†^**	37.6 **^a^**	7.3 **^b^**	40.8 **^a^**	0.0 **^b^**	46.8 **^a^**	77.5 **^c^**	0.0 **^§,b^**	4.2 **^b^**	48.5 **^a^**
9 Diet or “light” soft drinks **^†^**	8.5 **^a^**	3.3 **^a,b^**	20.4 **^a,c^**	0.0 **^b^**	27.8 **^c^**	6.3 **^a,b^**	0.0 **^§,b^**	1.2 **^b^**	4.2 **^a,b^**
10 Milk **^†^**	40.9 **^a^**	17.9 **^b^**	38.8 **^a,b^**	35.7 **^a,b^**	27.2 **^a,b^**	39.4 **^a^**	95.2 **^c^**	95.8 **^c^**	91.6 **^c^**
11 Flavoured milk **^†^**	36.7 **^a^**	18.7 **^b^**	32.7 **^a,b^**	3.3 **^c^**	18.3 **^b^**	15.6 **^b,c^**	43.2 **^a^**	41.9 **^a^**	18.6 **^b^**
12 Water **^†^**	35.5 **^a^**	56.1 **^b^**	62.2 **^b^**	50.7 **^a,b^**	55.0 **^b^**	68.1 **^b^**	99.2 **^c^**	47.3 **^a,b^**	3.0 **^d^**
13 Yoghurt **^†^**	35.2 **^a^**	4.9 **^b^**	28.6 **^a,c^**	13.6 **^b,c^**	37.3 **^a^**	70.0 **^d^**	13.6 **^b,c^**	15.6 **^b,c^**	29.3 **^a,c^**
14 Sweet snacks **^†^**	26.8 **^a^**	17.9 **^a,b^**	51.0 **^c^**	1.3 **^d^**	48.5 **^c^**	60.0 **^c^**	0.0 **^d^**	4.8 **^b,d^**	4.8 **^b,d^**
15 Salted snacks **^†^**	12.2 **^a^**	13.8 **^a,b^**	36.7 **^c^**	1.3 **^§,b,d^**	11.2 **^a,b^**	20.6 **^a,c^**	0.0 **^d^**	1.2 **^b,d^**	0.0 **^d^**
*Physical Activity-Related Characteristics*									
16 Availability of outside playgrounds or inside play areas where children can play during school breaks **^†^**	78.2 **^§,a^**	98.1 **^§,b^**	81.4 **^§,a,c^**	100.0 **^§,b^**	94.0 **^§,b,c^**	90.9 **^§,b,c^**	100.0 **^b^**	99.4 **^§,b^**	83.2 **^a,c^**
17 Provision of ≥60 min per week of physical education to pupils from participating classes **^†^**	98.5 **^§,a,b^**	95.4 **^§,a,b^**	100.0 **^§,a^**	88.2 **^§,b^**	98.8 **^a,b^**	100.0 **^§,a^**	95.8 **^§,a,b^**	91.5 **^§,a,b^**	100.0 **^a^**
*General Characteristic*									
18 Any initiatives/projects organized to promote a healthy lifestyle among pupils from participating classes **^†^**	87.0 **^§,a^**	42.3 **^§,b^**	88.4 **^§,a^**	93.5 **^§,a^**	95.2 **^a^**	50.4 **^§,b^**	58.1 **^§,b^**	92.0 **^§,a^**	84.4 **^a^**

Notes: * The country codes refer to the International Organization for Standardization (ISO) 3166-1 Alpha-3 country codes; **^†^** Statistically significant difference of proportions across the nine countries in COSI round 2 (2009/2010) (chi-squared test; *p* < 0.001); **^§^** Not all schools that returned a school form provided information for this characteristic (Table S3); **^a,b,c,d,e^** Within each characteristic (*i.e.*, by row), proportions that share the same letter superscript do not statistically significantly differ from each other (Marascuilo procedure); COSI, Childhood Obesity Surveillance Initiative; CZE, Czech Republic; GRC, Greece; HUN, Hungary; IRL, Ireland; LTU, Lithuania; LVA, Latvia; NOR, Norway; PRT, Portugal; SVN, Slovenia.

#### 3.1.2. School Nutrition Environment Score

The school nutrition environment scores, based on the five selected nutrition-related characteristics, are presented in Table 3. Based on the multiple comparisons of the mean scores across the countries, which was done for each round, it appeared that two non-overlapping clusters of countries could be determined at a score of 0.70 that were statistically significant from each other. The countries in the high-score cluster had a mean score of 0.70 or higher (letter superscripts a, c, d, e and h in round 1) and the countries in the low-score cluster had a mean score below 0.70 (letter superscripts b, f and g in round 1. None of the countries changed from one cluster to the other from round 1 to round 2. Two-way ANOVA tests showed a statistically significant interaction effect of country and round in the seven countries that participated in both rounds; hence, unpaired t-tests were performed and showed a statistically significant difference (*i.e.*, improvement) in mean scores between the two rounds in the Czech Republic, Portugal and Slovenia.

**Figure 1:**
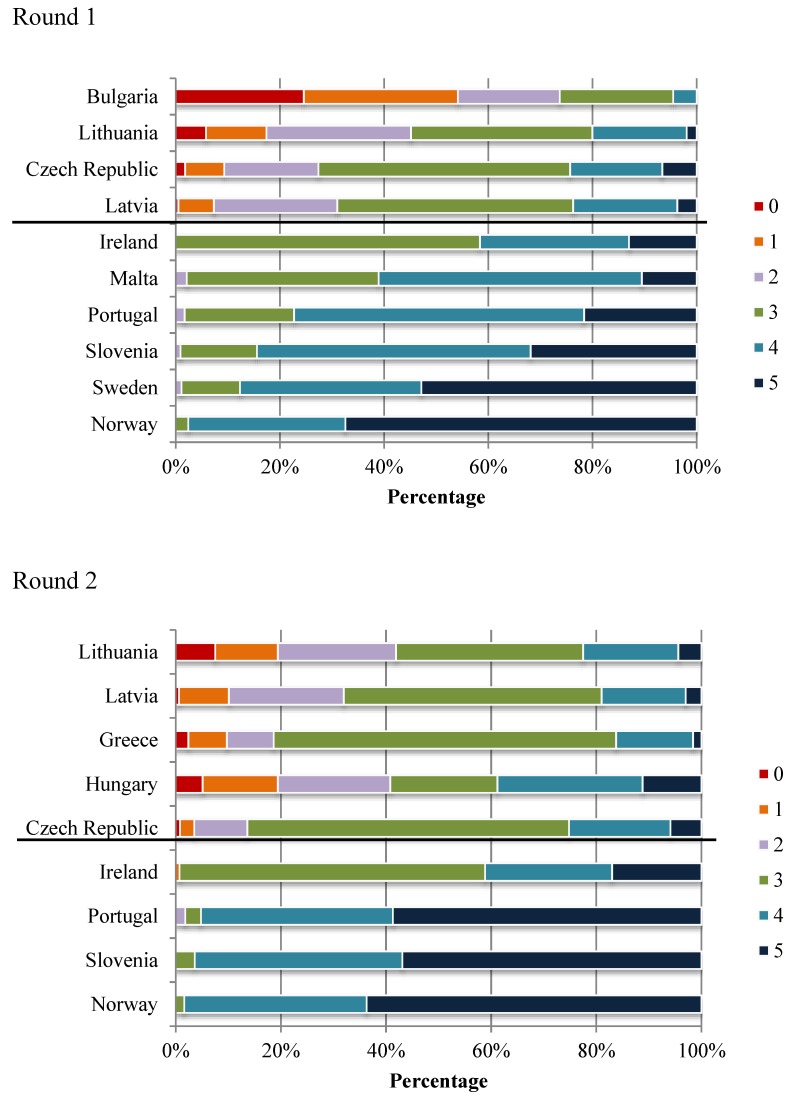
Distribution of schools in categories based on the number of characteristics graded as supportive (“five selected characteristics”) * in COSI rounds 1 (2007/2008) and 2 (2009/2010), by country (%) ^†^.

Notes: * Fresh fruit and milk can be obtained on the school premises and cold drinks containing sugar, sweet snacks or salted snacks cannot be obtained on the school premises; **^†^** Schools that provided information on all five selected nutrition-related school environment characteristics were included. The countries are ranked by score. The horizontal line distinguishes the low-score cluster (above the line; countries with a score of <0.70) from the high-score cluster (below the line; countries with a score ≥0.70); COSI, Childhood Obesity Surveillance Initiative.

Higher within-country variability in scores was detected for some countries, in particular in Bulgaria, Czech Republic Greece, Hungary, Latvia and Lithuania, compared with the other countries. This is clearly illustrated in Figure 1, which shows that both low-score and high-score schools were present in these six countries. Figure 1 also portrays that the median scores remained the same in all countries except Portugal and Slovenia in both rounds.

**Table 3:** School nutrition environment score characteristics of the schools that provided information on all five * selected characteristics in COSI rounds 1 (2007/2008) and 2 (2009/2010), by sampling approach and country.

Countries	Number of Schools		School Nutrition Environment Score	
	n		Mean ± SD (CV (%))	
	Round 1	Round 2	Round 1 ^†^	Round 2 ^†^
*Same Schools in Both Rounds*				
Ireland	154	153	0.71 ± 0.14 **^a^** (20.2)	0.71 ± 0.16 **^a^** (22.2)
Lithuania	155	160	0.51 ± 0.23 **^b^** (45.2)	0.52 ± 0.25 **^b^** (48.5)
Norway	126	124	0.93 ± 0.11 **^c^** (11.3)	0.92 ± 0.10 **^c^** (11.2)
Portugal **^§^**	176	167	0.79 ± 0.14 **^d,e^** (17.7)	0.90 ± 0.13 **^c^** (14.3)
*New Sample of Schools in Both Rounds*				
Bulgaria	179	–	0.30 ± 0.24 **^f^** (79.3)	–
Czech Republic **^§^**	548	882	0.58 ± 0.21 **^g^** (35.5)	0.63 ± 0.17 **^d^** (26.6)
Greece	–	123	–	0.57 ± 0.18 **^b,d^** (31.2)
Hungary	–	98	–	0.57 ± 0.28 **^b,d^** (49.3)
Latvia	190	169	0.58 ± 0.19 **^b,g^** (32.8)	0.56 ± 0.19 **^b^** (33.8)
Malta	95	–	0.74 ± 0.14 **^a,d^** (18.5)	–
Slovenia **^§^**	116	167	0.83 ± 0.14 **^e,h^** (16.7)	0.91 ± 0.11 **^c^** (12.5)
Sweden	89	–	0.88 ± 0.15 **^c,h^** (16.7)	–

Notes: * The five selected nutrition-related school environment characteristics were: fresh fruit, milk, cold drinks containing sugar, sweet snacks or salted snacks can be obtained on the school premises; **^†^** Statistically significant difference of mean scores across countries for the indicated round (one-way ANOVA; *p* < 0.0001); **^§^** Statistically significant difference of mean scores between the two rounds for the indicated country (unpaired t-test; *p* < 0.0001); **^a,b,c,d,e,f,g,h^** Within each round (*i.e.*, by column), scores that share the same superscript letter do not statistically significantly differ from each other (Games-Howell *post hoc* test); –, no participation; COSI, Childhood Obesity Surveillance Initiative; CV, coefficient of variation; SD, standard deviation.

#### 3.1.3. School BMI/A Z-Score

The choice for a minimum of 15 children with valid measurements resulted in an exclusion rate of 61% of the schools in Hungary. Lower but still substantial exclusion rates were found in Latvia (47%), Ireland (45%), Norway (39%) and Portugal (34%). The exclusion rates of schools in the other countries ranged from 1% to 22%.

Table 4 presents, for each country, the mean school BMI/A Z-score of the schools that measured the height and weight of at least 15 children. All mean Z-scores were positive, and the multiple comparisons show a high variability across the countries in both rounds whereby the highest scores were found in Greece, Ireland, Malta and Portugal. A large spread of scores within countries was also observed. Unpaired *t*-tests showed a statistically significant decrease in mean scores from the first to the second COSI round in Portugal only.

The country results of the linear regression analyses between the school BMI/A Z-scores and school categories that were based on the number of five nutrition-related characteristics graded as supportive are also given in Table 4. None of the country linear associations were statistically significant except in Sweden in round 1 (negative) and Norway in round 2 (positive). A statistically significant positive linear association was found for the total group of schools in both round 1 and round 2 (adjusted for country).

### 3.2. Discussion

#### 3.2.1. School Nutrition Environment

A large variability in school nutrition environment scores was found across countries whereby higher scores correspond to higher support for a healthy school nutrition environment. The low-score countries (Bulgaria, Czech Republic, Greece, Hungary, Latvia and Lithuania) graded on average less than 3 characteristics as supportive, and the high-score countries (Ireland, Malta, Norway, Portugal, Slovenia and Sweden) judged at least three out of the five graded characteristics supportive. We also observe a high variability in scores between schools (CV range: 11%−79%) within the same country, especially in the six low-score countries, where schools could be found in each quintile of the potential range of scores (between 0 and 1). None of the countries moved between clusters within the two-year observation period.

A number of methodological issues should be mentioned. Firstly, the COSI mandatory school form was not based on validated questionnaires, however, we pilot-tested a draft version of the COSI form in some of the participating countries prior to the first data collection in school year 2007/2008. This pilot resulted in 18 mandatory environment characteristics related to nutrition and physical activity.

**Table 4:** School BMI/A Z-score characteristics and linear regression analyses between school BMI/A Z-score and school category based on the number of characteristics graded as supportive * of primary schools ^†^ in COSI rounds 1 (2007/2008) and 2 (2009/2010), by sampling approach and country.

Countries	Number of Schools		School BMI/A Z-Score		Linear Regression with School Categories	
	n		Mean ± SD (CV (%))		α; ß (Probability F-Value)	
	Round 1	Round 2	Round 1 ^§^	Round 2 ^§^	Round 1	Round 2
*Same Schools in Both Rounds*						
Ireland	98	72 **^‼^**	0.54 ± 0.28 **^a,b^** (52.2)	0.50 ± 0.28 **^a,b^** (55.0)	0.46; 0.02 (0.688)	0.56; −0.01 (0.767)
Lithuania	135	136 **^‼^**	0.25 ± 0.28 **^c^** (111.7)	0.20 ± 0.38 **^c^** (191.6)	0.14; 0.03 (0.140)	0.16; 0.01 (0.667)
Norway	80	72	0.32 ± 0.27 **^c^** (84.7)	0.38 ± 0.33 **^a,d^** (86.2)	−0.10; 0.08 (0.197)	**−0.59; 0.18 (0.015)**
Portugal °	113	112	0.73 ± 0.32 **^d^** (43.7)	0.60 ± 0.34 **^b^** (56.2)	0.99; −0.05 (0.216)	0.59; 0.00 (0.972)
*New Sample of Schools in Both Rounds*						
Bulgaria	140	–	0.36 ± 0.45 **^c,e^** (123.7)	–	0.48; −0.05 (0.128)	–
Greece	–	114	–	1.02 ± 0.23 **^e^** (22.3)	–	1.11; −0.02 (0.329)
Hungary	–	38	–	0.29 ± 0.38 **^a,c,d^** (132.1)	–	0.24; 0.01 (0.734)
Latvia	100	92	0.23 ± 0.25 **^c^** (109.7)	0.27 ± 0.33 **^c,d^** (120.5)	0.22; 0.00 (0.924)	0.43; −0.05 (0.190)
Malta	74	–	0.59 ± 0.26 **^a^** (43.4)	–	0.45; 0.03 (0.468)	–
Slovenia	116	164 **^‼^**	0.46 ± 0.28 **^b,e^** (60.9)	0.40 ± 0.31 **^a,d^** (79.1)	0.70; −0.05 (0.223)	0.49; −0.02 (0.703)
Sweden	86	–	0.33 ± 0.21 **^c^** (64.4)	–	**0.73; −0.07 (0.019)**	–
Total countries	942	800	0.42 ± 0.34 (82.2)	0.47 ± 0.41 (87.5)	**0.29; 0.02 (0.0002) ^‡^**	**0.44; 0.09 (<0.0001) ^‡^**

Notes: * School categories were based on the number of selected nutrition-related school environment characteristics graded as supportive and ranged from 0 to 5, whereby supportive grading was defined as: fresh fruit and milk can be obtained on the school premises and cold drinks containing sugar, sweet snacks or salted snacks cannot be obtained on the school; **^†^** A school that had fewer than 15 children with valid measurements was excluded; **^§^** Statistically significant difference of mean scores across countries for the indicated round (one-way ANOVA; *p* < 0.0001); **^‼^** Classes that were sampled in round 2 to enrol the newly targeted 9-year-old age group were deleted; **^‡^** Multiple linear regression analysis was adjusted for country (coefficient for country: 0.01 in round 1 and −0.08 in round 2); ° Statistically significant difference of mean scores between the two rounds for the indicated country (unpaired t-test; *p* < 0.01); **^a,b,c,d,e^** Within each round, scores that share the same superscript letter do not statistically significantly differ from each other (Games-Howell *post hoc* test); –, no participation; α, constant term (y-intercept); ß, regression coefficient of the school category; significant F-values are shown in bold; BMI/A, body mass index-for-age; COSI, Childhood Obesity Surveillance Initiative; CV, coefficient of variation; SD, standard deviation.

Secondly, although not validated, the five selected nutrition-related characteristics were graded with the same weight (0 or 1), which is not an uncommon approach. For instance, Thomson *et al.* also did not give different weights to the yes/no responses of the “healthy/unhealthy” foodservice offerings items [27]. Like in our approach, they coded the availability of “healthy” offering items with a “1” and the availability of “unhealthy” offering items with a “0”, so that higher scores corresponded to higher occurrence of healthy practices. Turner and Chaloupka created a school food environment score for elementary schools by dividing the sum of “yes” responses for 16 items (scored either 0 or 1) by the number of valid items, then multiplying by 100. Each school’s score could range between 0 and 100 with higher scores indicating a healthier environment [37]. The pilot of the initial COSI school form also resulted in an additional set of school environment characteristics related to nutrition and physical activity that could be included on a voluntary basis by the countries (for example, the availability of vending machines, school canteen or cafeteria as well as the provision of fresh fruit, vegetables or milk for free, nutrition education or school bus transport). These optional characteristics were not included in the present analyses because they were not collected by all 12 countries. We also performed a score calculation based on 16 mandatory characteristics, which resulted in similar ranking of countries, except that Ireland would then be grouped in the low-score cluster, and Latvia and Lithuania in the high-score cluster (data not presented). The calculation of the score might have been improved by adding the optional characteristics to the mandatory part of the COSI form. This could be considered in future COSI rounds whereby it would be essential to sustain the balance between obtaining more information on a mandatory basis by all participating countries, keeping COSI as a surveillance system that is easy to implement with limited resources, and the added value of this additional information for the calculation of the school environment score.

Finally, COSI used a school-based cluster sampling approach to obtain a nationally representative sample of children, and thus we are confident that the calculated score can be considered representative for a country. Only the Czech Republic took another approach whereby the PSUs were paediatric clinics, which resulted in a higher number of schools linked to the children measured when compared to other countries.

One may speculate that the lower school nutrition environment score found in some countries is related to the absence or inadequate implementation of national policies. For instance, it is conceivable that a country had no formal national health promoting school policy at the time of the two COSI data collection rounds or that a policy existed but that it had not been fully implemented yet throughout the country. Unfortunately, not much information is available from literature. A European study done in 2013 among 43 Schools for Health in Europe member countries on the state of health promoting schools showed that 2%−10% of Czech schools, 20% of Lithuanian schools, 50% of Slovenian schools and 100% of Portuguese schools could be considered as health promoting schools [38]. In addition, sixteen (62%) of 26 National Coordinators that completed the questionnaire reported that their country has a formal national health promoting schools policy. Unfortunately the study reference did not list these 16 countries [38]. A Danish study suggests that organic food policies in schools may have the potential to support a healthier school food environment [39], and a study conducted in England observed considerable improvements in lunchtime food provision after the introduction of new compulsory standards for school food in 2009 [40].

Scholtens *et al.* [41] suggested that school size might be a factor playing a role in the high variation in scores between schools. They conducted a nationwide study among all Dutch secondary schools about the obesogenity of the school environment. Their findings suggest that vending machines containing soft drinks and/or sweets and candy bars, and facilities on or near school premises where students could be physically active were found less often in small schools (<500 students) than in large schools (>1000 students) [41]. Other factors might be the influence that school management has on the food supply offered [41], the presence of written school rules to restrict the consumption of savoury and sweet snacks [42] or school type (private *vs.* public) [27]. The COSI school form did not include questions on these four described factors, but they could be considered for inclusion in future rounds.

L’Abbé *et al.* have recently published a framework for assessing food environments in public sector settings whereby the school setting was used as a case study for its development [43]. We do not know whether this framework has already been used in European schools, and studies other than COSI done in Europe that described the school nutrition and physical activity environment in schools characteristics could only be identified for Belgium [42], the Netherlands [26,41,44] and Norway [45]. The five nutrition-related characteristics that have been selected to calculate a score are included in current EU policies [13,24] or have been considered in discussions to reduce the marketing of foods and non-alcoholic beverages to children [25]. Our findings suggest that the use of these five characteristics could already give a proxy indication of the level on which schools work on a healthy school nutrition environment. The combined absence of sugar-containing cold drinks, sweet snacks and salted snacks were more observed in high-score countries than in low-score countries (except in Slovenia where the availability of fresh fruit and the absence of sweet snacks and salted snacks led to a higher score). As a next step, local policy-makers should become familiar with further school details (including the voluntary COSI school form characteristics if collected by their country) before decisions can be made on which characteristics should be the target for further improvement of the healthy school nutrition environment. The involvement of the community and other local stakeholders (for instance, fruit suppliers) may be considered as well as other components that have been included in the WHO school policy framework for the implementation of the WHO global strategy on diet, physical activity and health [15], the WHO framework for health promoting schools [18,19,20] and the framework developed by L’Abbé *et al.* [43].

Longitudinal studies on the school nutrition and physical activity environment are scarce. The only European study we could identify was a nationwide Dutch study among secondary schools, which was able to show, over a period of four years, changes in both supportive and unsupportive nutrition or physical activity environment characteristics [26]. In this stage of the COSI-project, we could only compare the school nutrition environment over a two-year-interval, and a statistically significantly improvement in scores was observed in three countries. The improvement seen in the Czech Republic might be due to the implementation of both national [46] and local school projects on healthy nutrition [47], whereas the improvement seen in Portugal might be due to the rollout of various school projects throughout the country (for example the “Healthy Eating in School Health Program” [48]). The improvement in Slovenia, in particular the observed increase of the availability of fresh fruit in the schools (Table 1 and Table 2), might be related to the introduction of the school fruit and vegetables scheme during the school year 2009/2010 [49].

#### 3.2.2. School BMI/A Z-Score

A school BMI/A Z-score was calculated as the mean of the BMI/A Z-score of all children measured in that school. All country mean school values of the participating countries were positive and thus higher than the 2007 WHO growth reference [33]. A cluster of low-score countries (with a mean BMI/A Z-score value between the expected WHO reference value and 0.5 SD away from this reference median value) and a cluster of high-score countries (with a value more than 0.5 SD away from the 2007 WHO growth reference median) could be identified. Greece, Ireland, Malta and Portugal were grouped in the high-score cluster, thus in the group of countries with a high level of overweight. Over a period of two years, only Portugal showed a statistically significant decrease (*i.e.*, improvement) in scores, but none of the countries moved from one cluster to the other.

A methodological issue to point out is our choice of a minimum number of 15 measured children that was used for the estimation of a school mean BMI/A Z-score. Of course it would have been preferable to have set this minimum number higher to further increase the precision for the calculation of the school mean value. But for a relevant further increase of the precision, the number of children had to be increased substantially, and this would have led to an unacceptably low number of eligible schools. Most countries targeted only one age group and, following the COSI protocol, thus sampled one or two classes per school [7,8]. This resulted in 50% of schools in six countries that measured about 15–20 children or fewer (Table S1). With the minimum number set at 15 children, we encountered a school inclusion rate below 40% only in Hungary and a school inclusion rate of 53%−99% in the other countries. Because only 3% of Czech schools met this eligibility criterion, the Czech data, unfortunately, had to be excluded from these analyses. (The PSU in the Czech Republic was not schools but paediatric clinics, and only a few children from a paediatric clinic went to the same school).

In a normal distribution, the prevalence of overweight in countries with a mean BMI/A Z-score value of zero (thus similar to the growth reference median value) would be about 15%, and in countries with a mean BMI/A Z-score value of one (the WHO cut-off value for overweight in schoolchildren [33]) 50%. All country values were higher than zero but not higher than one (range: 0.20−1.02), meaning that the prevalence of overweight in the countries would be between 15% and 50% whereby the highest prevalence was found in Greece, Ireland, Malta and Portugal. Comparing these prevalence figures (thus based on school mean values) with other overweight prevalence studies using measured weight and height and based on the sample of children [50,51,52], it seems that our approach can give a proxy indication of the level of overweight in a country. However, we did not find statistically significant changes between rounds (except in Portugal) using school mean values as we have seen when using mean BMI/A Z-scores based on the entire sample of individual children [22].

The existence of school policies in schools was not studied in COSI, and information on socioeconomic factors (education and occupation of the parents) was only collected by a few countries through the COSI family form [7,8]. It is thus not clear whether these two factors could have influenced the high variability found in school BMI/A Z-scores between or within countries, as has been shown (but not consistently) by other studies. For instance, a European multicentre study [53] presented a heterogeneous association between socioeconomic factors and childhood overweight across different European regions, and a systematic review [54] suggests that some school policies may have been effective in improving the food environment and dietary intake in schools; but there was little assessment of their impact on BMI.

#### 3.2.3. Association between School Nutrition Environment and School BMI/A Z-Score

In our study, we used a school score approach to describe the nutrition environment and level of BMI across schools. For the individual countries we could not demonstrate an association between the school BMI/A Z-score and the school supportive level to a healthy nutrition environment, except for Norway and Sweden (Table 4). However, in these two high-score countries the range of scores was quite small (four or five characteristics were graded as supportive in 99% of the Norwegian schools and in 87% of the Swedish schools) and thus the regression results (different directions of association) should be interpreted with caution. Taking the school scores of all countries together, we found small but statistically significant positive associations between the school supportive level and the BMI level, indicating that at the moment of data collection the schools that were most active in creating a healthy nutrition environment were also the schools with the highest BMI/A Z-scores. Whether the nutrition policy at the schools was a consequence of the overweight and obesity level of the schools is unclear.

Several reviews have been done to assess the effectiveness of school-based interventions in reducing or preventing overweight and obesity among individual children and adolescents [55,56,57,58], and studies have been carried out to examine the association between environmental characteristics and BMI at the individual level [29,59]. We could not identify studies that used a school score approach to describe the level of overweight in schools.

It is generally known that overweight and obesity are multidimensional health problems resulting from a diverse set of factors and settings. For example, family environmental factors [60] or food outlets in the local environment [61] are potential contributors to child health and may have a stronger influence on developing excess body weight than school settings. In addition, the short time span of two years may have played a role, although a four-year longitudinal study also did not find an association between the introduction of competitive food sales in schools and children’s weight gain between fifth and eighth grades [62]. Furthermore, we did not investigate the time point when a policy to restrict or promote the availability of certain foods or beverages was introduced in a school (if it existed). Hence, we do not know whether the period between the introduction of this policy and the two COSI rounds was sufficiently long to observe an influence of a change in the school nutrition environment on the school BMI. Follow-up COSI rounds, as planned at a regular interval, may provide more conclusive answers provided that, at the same time, a time table of introduced new or modified policies is kept.

## 4. Conclusions

Some European countries have implemented more school policies that are supportive to a healthy nutrition environment than others. However, most countries with low school nutrition environment scores also host schools with supportive school environment policies, suggesting that a uniform school policy to tackle the “unhealthy” school nutrition environment has not been implemented at the same level throughout a country and may underline the need of harmonized school policies. The proxy indicator that we have applied may trigger policy-makers in the participating COSI countries to further elaborate on the school nutrition environment in their country. The indicator, when confirmed by other similar studies, could then assist countries in improving school interventions and monitoring their national policies targeting school settings and childhood obesity. Making the healthy choices available on their premises (e.g., presence of fresh fruit and milk; absence of cold drinks containing sugar, sweet snacks and salted snacks) schools may support pupils in adopting healthy eating habits.

## Supplementary Files

Supplementary File 1
