# Supplementary material for: WHO European Childhood Obesity Surveillance Initiative: School Nutrition Environment and Body Mass Index in Primary Schools
Source: Int J Environ Res Public Health. 2014 Oct 30;11(11):11261–85. doi: 10.3390/ijerph111111261 (PMC4245612; doi:10.3390/ijerph111111261)
Supplement: Supplementary File 1 [file ijerph-11-11261-s001.pdf]

WHO European Childhood Obesity Surveillance Initiative: School Nutrition Environment and Body Mass Index in Primary Schools

Table S1. School sampling characteristics and number of children measured per school in COSI rounds 1 (2007/2008) and 2 (2009/2010), by country.

Characteristics	BGR		CZE		GRC		HUN		IRL		LVA		LTU		MLT		NOR		PRT		SVN		SWE	
	COSI Data Collection Round																							
	**1**	**2**	**1**	**2 ***	**1**	**2**	**1**	**2**	**1**	**2** [†]	**1**	**2 ***	**1**	**2** [†]	**1**	**2**	**1**	**2** [†]	**1** [‡]	**2** [†,‡]	**1**	**2 ***	**1**	**2**
Targeted age groups	7	–	7	7	–	7, 9	–	7	7	7, 9	7	7	7	7, 9	6	–	8	8	7	7	6, 7, 8	6, 7, 8, 9	7, 8	–
Schools																								
Sampled (*n*)	184	–	NA [§]	NA [§]	–	150	–	164	498	192	193	174	161	164	95	–	131	131	185	185	118	167	220	–
Participated in weight and height measurements (*n*)	184	–	543 [§]	882 [§]	–	120	–	98	163	154	190	169	155	162	95	–	127	125	177	172	118	167	94	–
Completed a school form (*n*)	179	–	548 [§]	882 [§]	–	123	–	98	154	154	190	169	155	160	95	–	127	125	176	167	118	167	89	–
Children Measured in a School																								
Median (*n*)	19	–	1 [§]	1 [§]	–	41	–	12	18	24	16	15	29	55	29	–	23	20	18	21	99	90	40	–
Minimum (*n*)	2	–	1 [§]	1 [§]	–	1	–	1	2	2	2	1	8	12	3	–	2	1	1	2	33	7	11	–
Maximum (*n*)	43	–	29 [§]	31 [§]	–	169	–	34	32	60	99	115	79	170	104	–	108	85	50	55	172	228	131	–

Notes: –, no participation; BGR, Bulgaria; COSI, Childhood Obesity Surveillance Initiative; CZE, Czech Republic; GRC, Greece; HUN, Hungary; IRL, Ireland; LTU, Lithuania; LVA, Latvia; MLT, Malta; NA, not applicable; NOR, Norway; PRT, Portugal; SVN, Slovenia; SWE, Sweden; The country codes refer to the International Organization for Standardization (ISO) 3166-1 Alpha-3 country codes; ***** Country participated in both COSI rounds and a new sample of schools was included in round 2; [†] Country participated in both COSI rounds and the same schools as in round 1 were included in round 2; [‡] Data from four schools in Madeira, collected one year after the other Portuguese regions, were not taken into account in this paper; [§] The primary sampling unit were paediatric clinics and not primary schools. The schools of the measured children were, however, asked to complete the school form.

Table S2. Data collection period of the anthropometric measurements and the completion of the school form in COSI rounds 1 (2007/2008) and 2 (2009/2010), by country.

Country	Data Collection Period (Month/Year)			
	Anthropometric Measurements		Completion of School Form	
	Round 1	Round 2	Round 1	Round 2
Bulgaria	03/08–06/08	–	NA	–
Czech Republic	01/08–12/08	01/10–12/10	01/08–09/09	01/10–03/11
Greece	–	11/10–03/11	–	11/10–03/11
Hungary	–	04/10–06/10	–	04/10–06/10
Ireland	04/08–06/08	10/10–11/10	04/08–09/08	10/10–11/10
Latvia	02/08–03/08	03/10–04/10	02/08–03/08	03/10–04/10
Lithuania	04/08–05/08	02/10–05/10	03/08–05/08	03/10–05/10
Malta	04/08–06/08	–	04/08–08/08	–
Norway	09/08–11/08	09/10–12/10	09/08–11/08	09/10–12/10
Portugal	05/08–06/08	04/10–12/10	05/08–07/08	05/10–12/10
Slovenia	04/08–04/08	04/10–04/10	12/08–12/08	05/10–05/10
Sweden	03/08–06/08	–	03/08–11/08	–

Notes: –, no participation; NA, not available.

Table S3. Number of schools that completed a school form in COSI rounds 1 (2007/2008) and 2 (2009/2010), by total number of mandatory school environment characteristics for which information was provided and country.

Total Number of Mandatory School Environment Characteristics for Which Information was Provided	BGR		CZE		GRC		HUN		IRL		LVA		LTU		MLT		NOR		PRT		SVN		SWE	
	COSI Data Collection Round																							
	1 [a]	2	1 [b]	2 [c]	1	2 [d]	1	2 [e]	1	2 [f]	1	2 [g]	1 [h]	2 [i]	1	2	1 [j]	2 [k]	1 [l]	2 [m]	1 [n]	2	1 [o]	2
	Number of Schools (*n*)																							
<13	0	–	0	0	–	0	–	0	0	0	0	0	0	0	0	–	0	0	0	0	0	0	0	–
13	0	–	0	0	–	0	–	0	0	0	0	0	0	0	0	–	1	1	0	0	0	0	0	–
14	0	–	0	0	–	0	–	0	0	0	0	0	0	0	0	–	0	0	0	0	0	0	0	–
15	0	–	109	381	–	14	–	0	0	0	0	0	1	3	0	–	0	0	0	0	0	0	0	–
16	5	–	6	20	–	1	–	1	0	1	0	1	9	9	0	–	0	0	6	2	0	0	47	–
17	13	–	14	33	–	7	–	4	0	10	0	2	14	27	0	–	7	6	14	6	5	0	5	–
18	161	–	419	448	–	101	–	93	154	143	190	166	131	121	95	–	119	118	156	159	113	167	37	–

Notes: –, no participation; BGR, Bulgaria; COSI, Childhood Obesity Surveillance Initiative; CZE, Czech Republic; GRC, Greece; HUN, Hungary; IRL, Ireland; LTU, Lithuania; LVA, Latvia; MLT, Malta; NOR, Norway; PRT, Portugal; SVN, Slovenia; SWE, Sweden; The country codes refer to the International Organization for Standardization (ISO) 3166-1 Alpha-3 country codes; [a] Information was not provided by some schools on the characteristics “availability of outside playgrounds or inside play areas where children can play during school breaks” (*n* = 2), “provision of physical education to pupils from participating classes” (*n* = 7) and “any initiatives/projects organized to promote a healthy lifestyle among pupils from participating classes” (*n* = 14); [b] Information was not provided by some schools on the characteristics “availability of outside playgrounds or inside play areas where children can play during school breaks” (*n* = 117), “provision of physical education to pupils from participating classes” (*n* = 120) and “any initiatives/projects organized to promote a healthy lifestyle among pupils from participating classes” (*n* = 116); [c] Information was not provided by some schools on the characteristics “availability of outside playgrounds or inside play areas where children can play during school breaks” (*n* = 395), “provision of physical education to pupils from participating classes” (*n* = 409) and “any initiatives/projects organized to promote a healthy lifestyle among pupils from participating classes” (*n* = 412); [d] Information was not provided by some schools on the characteristics “availability of outside playgrounds or inside play areas where children can play during school breaks” (*n* = 17), “provision of physical education to pupils from participating classes” (*n* = 15) and “any initiatives/projects organized to promote a healthy lifestyle among pupils from participating classes” (*n* = 19); [e] Information was not provided by some schools on the characteristics “availability of outside playgrounds or inside play areas where children can play during school breaks” (*n* = 1), “provision of physical education to pupils from participating classes” (*n* = 2) and “any initiatives/projects organized to promote a healthy lifestyle among pupils from participating classes” (*n* = 3); [f] Information was not provided by some schools on the characteristics “salted snacks can be obtained on the school premises” (*n* = 1), “availability of outside playgrounds or inside play areas where children can play

during school breaks" (*n* = 8), "provision of physical education to pupils from participating classes" (*n* = 2) and "any initiatives/projects organized to promote a healthy lifestyle among pupils from participating classes" (*n* = 1); [g] Information was not provided by some schools on the characteristics "availability of outside playgrounds or inside play areas where children can play during school breaks" (*n* = 2) and "any initiatives/projects organized to promote a healthy lifestyle among pupils from participating classes" (*n* = 2); [h] Information was not provided by some schools on the characteristics "availability of outside playgrounds or inside play areas where children can play during school breaks" (*n* = 2), "provision of physical education to pupils from participating classes" (*n* = 9) and "any initiatives/projects organized to promote a healthy lifestyle among pupils from participating classes" (*n*= 24); [i] Information was not provided by some schools on the characteristics "availability of outside playgrounds or inside play areas where children can play during school breaks" (*n* = 17), "provision of physical education to pupils from participating classes" (*n* = 14) and "any initiatives/projects organized to promote a healthy lifestyle among pupils from participating classes" (*n* = 23); [j] Information was not provided by some schools on the characteristics "cold drinks without sugar can be obtained on the school premises" (*n* = 1), "cold drinks containing sugar can be obtained on the school premises" (*n* = 1), "hot drinks without sugar can be obtained on the school premises" (*n* = 1), "hot drinks containing sugar can be obtained on the school premises" (*n* = 1), "diet or 'light' soft drinks can be obtained on the school premises" (*n* = 1), "provision of physical education to pupils from participating classes" (*n* = 1) and "any initiatives/projects organized to promote a healthy lifestyle among pupils from participating classes" (*n* = 6); [k] Information was not provided by some schools on the characteristics "cold drinks without sugar can be obtained on the school premises" (*n* = 1), "cold drinks containing sugar can be obtained on the school premises" (*n* = 1), "hot drinks without sugar can be obtained on the school premises" (*n* = 1), "hot drinks containing sugar can be obtained on the school premises" (*n* = 1), "diet or 'light' soft drinks can be obtained on the school premises" (*n* = 1), "provision of physical education to pupils from participating classes" (*n* = 5) and "any initiatives/projects organized to promote a healthy lifestyle among pupils from participating classes" (*n* = 1); [l] Information was not provided by some schools on the characteristics "availability of outside playgrounds or inside play areas where children can play during school breaks" (*n* = 4), "provision of physical education to pupils from participating classes" (*n* = 14) and "any initiatives/projects organized to promote a healthy lifestyle among pupils from participating classes" (*n* = 8); [m] Information was not provided by some schools on the characteristics "availability of outside playgrounds or inside play areas where children can play during school breaks" (*n* = 2), "provision of physical education to pupils from participating classes" (*n* = 3) and "any initiatives/projects organized to promote a healthy lifestyle among pupils from participating classes" (*n* = 5); [n] Information was not provided by some schools on the characteristics "fresh fruit can be obtained on the school premises" (*n* = 2), "diet or 'light' soft drinks can be obtained on the school premises" (*n* = 1), "availability of outside playgrounds or inside play areas where children can play during school breaks" (*n* = 1) and "any initiatives/projects organized to promote a healthy lifestyle among pupils from participating classes" (*n* = 1); [o] Information was not provided by some schools on the characteristics "flavoured milk can be obtained on the school premises" (*n* = 1), "availability of outside playgrounds or inside play areas where children can play during school breaks" (*n* = 1), "provision of physical education to pupils from participating classes" (*n* = 47) and "any initiatives/projects organized to promote a healthy lifestyle among pupils from participating classes" (*n* = 50).

© 2014 by the World Health Organization; licensee MDPI, Basel, Switzerland. This article is an open access article distributed under the terms and conditions of the Creative Commons Attribution IGO license (http://creativecommons.org/licenses/by/3.0/igo).
